# Potential Improvement in Rehabilitation Quality of 2019 Novel Coronavirus by Isometric Training System; Is There “Muscle-Lung Cross-Talk”?

**DOI:** 10.3390/ijerph18126304

**Published:** 2021-06-10

**Authors:** Hadi Nobari, Mohamad Fashi, Arezoo Eskandari, Jorge Pérez-Gómez, Katsuhiko Suzuki

**Affiliations:** 1 Department of Physical Education and Sports, University of Granada, 18010 Granada, Spain; 2 HEME Research Group, Faculty of Sport Sciences, University of Extremadura, 10003 Cáceres, Spain; jorgepg100@gmail.com; 3 Department of Exercise Physiology, Faculty of Sport Sciences, University of Isfahan, Isfahan 81746-7344, Iran; 4 Department of Biological Sciences in Sports, Faculty of Sports Science and Health, Shahid Beheshti University, Tehran 198396-3113, Iran; 5 Department of Exercise Physiology, Faculty of Physical Education and Sports Science, Tehran University, Tehran 141793-5840, Iran; A.eskandari_1988@yahoo.com; 6 Faculty of Sport Sciences, Waseda University, Saitama 359-1192, Japan

**Keywords:** COVID-19, immune response, chronic diseases, exercise, oxidative stress, anti-inflammatory treatment, fibroblast growth factor 21, cytokines, myokines

## Abstract

The novel Coronavirus Disease 2019 (COVID-19) crisis is now present in more than 200 countries. It started in December 2019 and has, so far, led to more than 149, 470,968 cases, 3,152,121 deaths, and 127,133,013 survivors recovered by 28 April 2021. COVID-19 has a high morbidity, and mortality of 2%, on average, whereas most people are treated after a period of time. Some people who recover from COVID-19 are left with 20 to 30% decreased lung function. In this context, exercise focused on skeletal muscle with minimal lung involvement could potentially play an important role. Regular exercise protects against diseases associated with chronic low-grade systemic inflammation. This long-term effect of exercise may be ascribed to the anti-inflammatory response elicited by an acute bout of exercise, which is partly mediated by muscle-derived myokines. The isometric training system seems to have this feature, because this system is involved with the skeletal muscle as the target tissue. However, no studies have examined the effect of exercise on the treatment and recovery of COVID-19, and, more importantly, “muscle–lung cross-talk” as a mechanism for COVID-19 treatment. It is suggested that this theoretical construct be examined by researchers.

## Dear Editor

Coronavirus Disease 2019 (COVID-19) is a new infectious disease caused by the coronavirus 2 that causes extreme acute respiratory syndrome (SARS-CoV-2). The majority of COVID-19 patients have mild to moderate symptoms, with about 15% progressing to extreme pneumonia and about 5% developing acute respiratory distress syndrome. The COVID-19 crisis is now present in more than 200 countries. It started in December 2019 and has, so far, led to more than 149,470,968 cases, 3,152,121 deaths, and 127,133,013 survivors recovered by 28 April 2021. COVID-19 is too new to have enough information about it; therefore, it may become an unprecedented pandemic. It has also spread to many Asian, American, and European countries. Causing respiratory tract infection and fibrosis, COVID-19 could result in morbidity and mortality, especially in those with impaired immune systems or a lack of existing immunity to the new virus [1]. In severe cases of COVID-19 infection, the virus can enter the blood stream and infect endothelial and other target cells in the kidneys, esophagus, bladder, ileum, heart tissues, and central nervous system; multiple organ failure associated with hyper activation of the immune system is observed. Patients with COVID-19 infection in critical condition often have high systemic inflammatory parameters, including levels of creative protein and cytokines (e.g., interleukin 6 (IL-6), IL-8, tumor necrosis factor alpha (TNF-α), and etc.) [2]. In general, cytokine formation, inflammation, cell death, and other pathophysiological processes are linked to respiratory viral infections, which may be linked to a redox imbalance or oxidative stress. NADPH oxidase-2 (NOX-2) is overexpressed in hospitalized COVID-19 patients, resulting in increased oxidative stress, according to Violi et al. Other authors have found that blocking NOX-2 enhances disease phenotypes in macrophages by reducing oxidative stress, which is consistent with these findings [3].

Endothelial cells can mobilize NOX proteins in response to pro-inflammatory cytokines (e.g., IL-1, IL-6, and TNF-α) and other agonists [4]. This contributes to local oxidative stress, which leads to endothelial dysfunction. NOX proteins may be mobilized by endothelial cells, leading to local oxidative stress and, as a result, endothelial dysfunction [5]. This causes muscle damage, either directly or indirectly, through oxidative damage to biomolecules and the activation of pro- or anti-inflammatory cytokines [6]. There is, however, insufficient information on COVID-19 and exercise. Fever, heavy pneumonia, ribonucleic acid (RNA) aemia, as well as the occurrence of ground-glass opacities and acute cardiac injury are some of the markers associated with COVID-19. In patients with COVID-19, substantial high blood levels of cytokines and chemokines (chemokine ligand (CCL) 2 and CCL5 and their receptors (CCR) 2 and CCR5) have been found. Chemokines have biological effects, such as inducing host immune cells to travel to the site of infection, controlling lymphocyte and other leukocyte transmission via peripheral lymph tissues, and promoting the growth of non-lymphatic organs [7]. The “cytokine storm” causes a pro-inflammatory climate, which is linked to significant tissue damage and contributes to COVID-19 patients’ fatal outcomes. The relation between inflammation and oxidative stress has been well established [8]. Nuclear factor-kappa B (NF-κB) and Toll-like receptor 4 (TLR4) signaling pathways, which are primarily triggered by viral pathogens such as SARS-CoV-2, can intensify the host inflammatory response, eventually leading to acute lung injury. TNF-α and IL-6, which are formed by skeletal muscle, T-cells, macrophages, and natural killer cells, are elevated as part of this response [9]. This disease, which is followed by a cytokine storm, affects a variety of tissues, most notably the lungs, resulting in acute lung injury of varying degrees. The cause of some patients’ rapid progression from acute respiratory distress syndrome to septic shock, accompanied by multiple organ failure and death, is cytokine storm. Acute lung injury and sepsis-induced multiple organ failure, two of the most common causes of morbidity and mortality in serious illness, have also been linked to oxidative stress. As a result, oxidative stress has long been a promising therapeutic target in serious illness, and antioxidants have long been studied in critically ill patients. Reactive oxygen species (ROS) are important mediators of the inflammatory responses, and different levels of exercise have mediate effects on ROS development. The inside membrane of the mitochondria is a source of ROS. Different levels of oxidative stress are caused by different levels of severity, volume of training, and exposure to a stressful setting [6]. Hence, antioxidants have anti-inflammatory properties and can be useful in the treatment of cytokine storm [10]. In addition, the transcription factor nuclear factor erythroid 2-related factor 2 (Nrf2) is responsible for the adaptation of cells under electrophilic or oxidative stresses [11]. Under normal conditions, Nrf2 is located in the cytoplasm bound to its inhibitor Keap1, which targets Nrf2 for ubiquitination and subsequent degradation. In the presence of electrophiles or ROS, the Keap1-Nrf2 complex dissociates and Nrf2 migrates to the nucleus, where it stimulates transcription of the target genes with antioxidant response element sequences in their promoters [11,12].

Exercise’s anti-inflammatory effects have been studied through three different mechanisms: (1) a decrease in visceral fat mass; (2) increased synthesis and release of anti-inflammatory cytokines (e.g., interleukin 1 receptor antagonist (IL-1ra), interleukin 4 (IL-4), interleukin 10 (IL-10), interleukin 11 (IL-11), and interleukin 13 (IL-13)), by contracting skeletal muscle, such as myokines; and (3) TLR expression on monocytes and macrophages is reduced as a result [13]. In addition, exercise is a way in which it is possible to induce Nrf2 migrates to the nucleus, and this stimulates antioxidant response element and pro/anti-inflammatory balance [14].

Several studies have demonstrated that physical fitness and moderate-intensity training also have reverse correlations with risks of disease and premature fatality [15,16], which can be that immune response function and improved types of immune markers can be increased by exercise in some diseases. These diseases can include low-grade inflammation, hypertension, stroke, osteoporosis, cancer (e.g., colon, lung, stroke, and breast), chronic infectious disease, metabolic syndrome, cardiovascular disease, type 2 diabetes mellitus, cognitive impairment and obesity [6,17,18].

**Figure 1:**
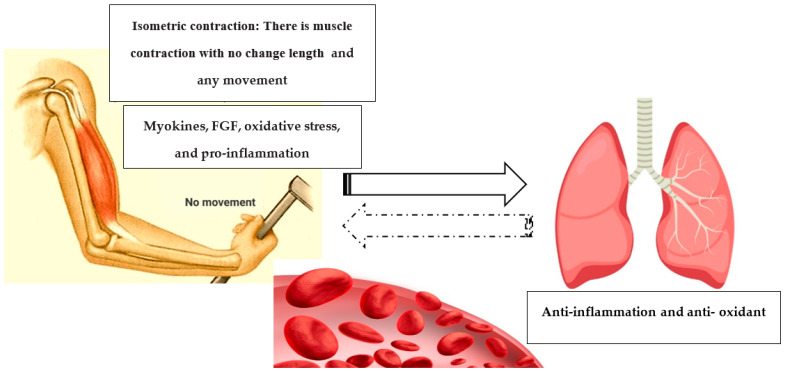
The relationship between isometric training system exercise and the release of myokines and FGF as anti-inflammatory effects that suppress inflammatory reactions (muscle–lung cross-talk). FGF: fibroblast growth factor.

It is safe to exercise during the COVID-19 epidemic in healthy individuals, taking the necessary precautions, such as exercising at home. After COVID-19 disease, due to the spread of infection, the patients will not be able to exercise [16,19]. COVID-19 mortality is on average 2%, whereas most people are treated after a specified period. Some people who recover from COVID-19 are left with 20 to 30% less lung function, and gasping for breath when they walk quickly. In this context, exercise could potentially play an important role during recovery from COVID-19. Due to respiratory tract infection, performing aerobic exercises for recovered patients will be extremely difficult. Therefore, exercise focused on skeletal muscle with minimal lung involvement should be a priority [20]. The isometric training system seems to have this feature. Isometric training is used in the rehabilitation and physical preparation of athletes, patients, and the general public. Isometric training refers to muscle contraction where the muscle-tendon unit remains at a constant length. An example of this is pushing against an immovable object such as a wall. Suitable training using this system consists of 5–10 repetitions of 5 s per contraction, 5 days a week. Isometric contractions have a number of benefits, according to some sources; (i) in rehabilitative environments, isometric training allows for a precise application of force within pain-free joint angles; (ii) since the maximal isometric force is greater than that of concentric contractions, isometric training can help to trigger force overload which is associated with neuromuscular adaptations; and (iii) a professional who is familiar with a sport’s physical demands will be able to use isometric training to target particular weak points in a range of motion, which can help with success and injury prevention [21,22,23]. By altering excitatory and inhibitory functions in the corticomotor pathways, isometric contractions may also provide an acute analgesic effect and allow for painless dynamic loading. Isometric contractions are also a highly accurate way to measure and monitor changes in force production. The isometric training method appears to aid immune system resilience by reawakening antioxidant defenses such as the glutathione system. Without the involvement of other antioxidant systems, the glutathione system could provide a primary protection against ROS produced during the performed isometric system, preventing oxidative damage to cellular lipids [21,24,25]. According to self-reported findings by four of the patients recovered from COVID-19, doing home isometric exercises has a great impact on the speed of return to normal life during post-illness recovery (Kashan, Esfahan, Iran: unpublished observation). The isometric training system exercises the skeletal muscle as the target tissue, where it can induce useful adaptations. These adaptations include, in part, the anti-inflammatory and antioxidant effects of exercise and could play an important role during the pandemic. Myokines are cytokines or peptides synthesized and released by myocytes and immune cells in muscle tissue in response to muscular contractions [26]. Myokines are implicated in the autocrine regulation of metabolism in muscles as well as in the para/endocrine regulation of other tissues and organs including the adipose tissue, liver, and brain and lung through their receptors “muscle–lung cross-talk”. For example, decreased levels of irisin, a skeletal muscle cell-derived myokine, are related to reduced lung function [27]. Irisin secretion was found to be linked to physical activity in a previous study. Irisin could play a role in oxidant stress inhibition through an oxidative-restraint pathway involving Nrf2, which leads to cell apoptosis [28]. Furthermore, by upregulating PPAR expression and suppressing inflammatory cytokine levels, fibroblast growth factor 21 (FGF-21) reduces pulmonary hypertension and suppressing inflammatory cytokine levels. FGF is a group of 18 polypeptides with biochemical properties that are structurally and functionally significant. To regulate glycolipid metabolism, enhance insulin resistance, prevent liver disease, and promote the browning of white adipose tissue, FGF-21 can enter the blood in autocrine, endocrine, and paracrine patterns [29]. Exercise can increase FGF-21 secretion and expression, effectively controlling brown adipose tissue activation and white adipose tissue browning to aid fat loss. These improvements can be linked to an increase in cardiorespiratory health [30,31]. This mechanism can be seen in Figure 1.

The benefits of pulmonary rehabilitation are well known and the existing programs could be used as one of the rehabilitation referral pathways for COVID-19 survivors who present with symptoms and/or impairments in physical function. The main component of pulmonary rehabilitation programs is exercise training, which includes aerobic and/or resistance training, and these exercises have been demonstrated to decrease the negative effects that prolonged sedentary behavior and inactivity during a hospitalization period have on physical function [32]. The basic mechanism of these adaptations about COVID-19 is not fully understood. In addition, to date, no studies have examined the effect of exercise on the recovery of COVID-19, and more importantly “muscle–lung cross-talk” as a mechanism for COVID-19 rehabilitation. We encourage exercise physiology and immunology researchers to examine the theoretical constructs presented here.

## Data Availability

Not applicable.
